# State of the art in on-line techniques coupled to flow injection analysis FIA/on-line- a critical review

**DOI:** 10.1155/S1463924690000219

**Published:** 1990

**Authors:** R. Puchades, A. Maquieira, J. Atienza, M. A. Herrero

**Affiliations:** Department of Chemistry Politechnic University of Valencia Camino de Vera, 14 Valencia 46022 Spain

## Abstract

Flow injection analysis (FIA) has emerged as an increasingly used laboratory tool in chemical analysis. Employment of the technique for on-line sample treatment and on-line measurement in chemical process control is a growing trend. This article reviews the recent applications of FlA. Most papers refer to on-line sample treatment. Although FIA is very well suited to continuous on-line process monitoring, few examples have been found in this areamost of them have been applied to water treatment or fermentation processes.


Journal of Automatic Chemistry, Vol. 12, No. 4 (July–August 1990), pp. 163–173

### State of the art in on-line techniques coupled to flow injection analysis FIA/on-line – a critical review

**R. Puchades, A. Maquieira, J. Atienza and M. A. Herrero**

*Department of Chemistry, Politechnic University of Valencia, Camino de Vera, 14, 46022-Valencia, Spain*

*Flow injection analysis (FIA) has emerged as an increasingly used laboratory tool in chemical analysis. Employment of the technique for on-line sample treatment and on-line measurement in chemical process control is a growing trend. This article reviews the recent applications of FIA. Most papers refer to on-line sample treatment. Although FIA is very well suited to continuous on-line process monitoring, few examples have been found in this area – most of them have been applied to water treatment or fermentation processes.*

### Introduction

The goal of process analytical chemistry is to supply quantitative and qualitative information about a chemical process. Such information can be used not only to monitor and control a process, but also to optimize its efficient use of energy, time and raw materials.

Process chemistry generally falls under the umbrella term of 'process control'. However, process chemistry has three different functions: process analysis, process monitoring and process control [1]. Process monitoring is the determination of the analyte(s) of interest in order to evaluate what is happening.

On-line refers to an analytical system which takes a sample directly from the process stream and immediately begins the determination of the analyte [2]. An automated sampling system is used to extract the sample, condition it and present it to an analytical instrument for measurement [3]. It is possible to subdivide the on-line techniques into two categories [4]: intermittent methods that require injection of a portion of the sample stream into the instrument; and continuous methods that permit the sample to flow continuously through the instrument. Continuous on-line process analysis is the first technique to offer true real-time capability.

Different literature references mention the need for on-line analysis [5–8], as well as the corresponding theoretical remarks [9]. Nichols [10] introduces readers to on-line process analysis by focusing primarily on existing analysers or sensor technology.

On-line process analysers using spectrometric techniques are employed in industry for a variety of applications, most of them involving the selective measurement of the concentration of one or more components of interest. Measurement is primarily made in the gaseous phase, but instruments are also available and have been successfully used for measurement in the liquid and solid phases. Many of these applications and some of the commercially available equipment are described by Clevett [11].

In relation to the development of commercially available on-line process analysers, there are two areas of recent development: on-line *Fourier Transform Infrared Spectroscopy* (FTIR) and *Optosensors*. However, the field of spectroscopy has probably seen a greater development in the last decade – as a result of advances in technology – than any other analytical chemistry.

Most process plants in operation today are still primarily controlled by monitoring operational physical variables, such as temperature, pressure, flow and liquid level. The higher failure rate and the higher demands on maintenance of on-line analysers are mainly due to the greater complexity of the equipment [12]. In spite of the difficulties encountered, there is a growing interest in process analysers with chemical reactions.

The majority of standard test methods required for laboratory measurement of water quality parameters make use of traditional wet chemical techniques. Over the past 20 years, automatic analysers have been developed and used successfully for standard tests in the laboratory. The first attempts to develop on-line wet chemical analysers took the form of converting automatic laboratory analysers to process use [13].

On-line wet chemical analysers are extremely versatile and can be used to measure a wide range of components covering a variety of industrial applications, particularly those in water and waste-water treatment processes. Manufacturers present a wide range of on-line, wet chemical analysers based on the colorimetric, titrimetric, or potentiometric principle of measurement and designed on the same modular basis [14].

Since the number of different component analysers available on the market is quite limited, and this situation is not expected to improve in the near future, it is very attractive to focus on flexible modular systems that can easily be adapted to achieve the desired type of analysis [15]. It is within this framework that such versatile methods as air-segmented continuous flow analysis (CFA) and flow injection analysis (FIA) come into the picture.

### Flow injection analysis

The continuous analytical methods involve the constant circulation of stream through the system, wherein the sample is introduced by aspiration or injection and

0142–0453/90 $3.00 © 1990 Taylor & Francis Ltd.

subsequently led to the detection cell. The methods in this group can be further classified [16] according to whether they use air bubbles (SFA) [17,18] or not (UFA), to avoid carry-over between successively injected samples. Unsegmented flow methods can be divided into those injecting (FIA) [19] or aspirating (CCFA) [20] the sample into the system.

Flow injection analysis (FIA) has many features that should make it a valuable technique for on-line process control and may be considered as an on-line alternative to manual wet chemical analysis [21].

The advantages of using FIA for process monitoring include: rapid change of methods, large throughput, real-time data acquisition, and the possibility of performing matrix modification [22]. The last two advantages are the most pertinent to process monitoring. Real- or near real-time data acquisition is critical to the efficiency of the process control system [23]. Matrix modification is important in the development of the analytical method. The usual process stream has a matrix which makes it difficult to selectively measure the analyte. An FIA system can incorporate matrix modification techniques which minimize or eliminate matrix problems [24]. The FIA system can be used to automate existing methods or to slightly modify and improve them. More importantly, the FIA system can utilize selectivity enhancement, kinetic discrimination, and matrix optimization to create new methods which are not possible in the batch or segmented continuous flow systems [1]. On the other hand, flow injection systems are serious candidates for a new generation of chemical on-line analysers because there is a growing interest in instruments that combine versatility with the possibility of attaining high sampling frequencies [9]. For real on-line applications the instrument and its component parts have to meet the highest standards with respect to reliability and maintenance [19].

FIA will be more widely used as new technologies are added. First is the capability to perform on-stream extractions. Second is the incorporation of a chromatographic step. Third is the inclusion of sophisticated multichannel, rapid scan spectroscopic detectors to provide a more robust single-component analysis or simultaneous multicomponent analysis [4].

Concentration of starting materials and products in batch processes often change over a range of three or more decades through the production. On-line monitoring of these processes should cover the whole range but the precision needed in the different ranges depends on the task of the analyser. FIA offers different simple techniques to fulfil such requirements. This makes the FIA technique useful for on-line analysers in pilot and production plants [25]. However, only a few relevant articles have been published about this highly competitive area of on-line process control [26]. Lázaro *et al.* [27] have summarized the possibilities of FIA in on-line process control.

To carry out this review, only those articles whose title and/or summary contain the keywords 'on-line' or 'continuous monitoring' and 'FIA' have been included.

### Sampling systems

Sampling is a critical problem in developing on-line measuring techniques and plays a key role. FIA will not improve the sampling capabilities of an analytical system. However, it will provide the means to acquire larger data sets, which will permit greater statistical confidence in the results [1]. In FIA, the injection valve is certainly the weakest part. A flow-injection analyser for on-line process control should be constructed in such a way that maintenance and replacement of valves van be easily accomplished.

Although FIA is very well suited, in principle, for continuous on-line monitoring, very few examples can be found in the literature. Most of them apply to the field of water quality and pollution monitoring [28,29]. Hardly any example so far deals with real process analysis. This is at least partly due to problems associated with the design and construction of adequate sampling and sample conditioning systems [30].

Some important requirements that the sampling system has to meet have been described by van der Linden [15] and Valcarcel and Gallego [31]. Nichols [10] provides an interesting discussion of sample systems and points towards one of the major problems in implementing a process analyser. Several authors in other reviews deal with this problem [32,33]. A review that includes a study of sampling systems available for on-line analysers used in quality control is presented by van der Linden [34].

### *Reversed FIA*

In normal FIA the solution containing the analyte is injected into an inert element, or into an eluent containing an excess of a reagent, and is determined by means of a suitable detector either directly or as a derivative [35]. In general, reagent consumption in normal FIA is generally 1 ml/min. This can be excessive when working continuously (around the clock), especially when expensive reagents have to be used. An alternative to this problem is the possibility of reversed FIA [36].

In reversed FIA (rFIA) the position of sample and reagent solutions are reversed; the reagent solution is injected into the sample stream, which is now the eluent [37]. The fact that the reverse FIA method can be particularly advantageous in control situations for determinations in cheap, plentiful sample solutions has been previously pointed out [38]. The main advantages of rFIA are economy in reagents and waste disposal and the possibility of making several different determinations on the same sample solution stream, for example by the injection of different reagents [39].

Amperometric methods of this type have been developed for the indirect determination of aromatic amines by on-line diazotisation reactions involving on-line rFIA formation of nitrosyl bromide [40], and on-line brominations with on-line rFIA formation of bromine [3]. An iodimetric method for the determination of sulphite by on-line rFIA was used by Fogg [35]. On-line sulphate monitoring in water by rFIA was described by van Staden [41]. Many other examples are described in the literature [42–44].

### FIA/on-line sample treatment

The use of ion exchange columns for trace preconcentration and interferent removal is one of the most widely used on-line technique in FIA systems.

Olsen *et al.* [45] showed that on-line trace enrichment in a flow injection system is extremely efficient. They used columns with Chelex-100 in a flow system connected to an atomic absorption spectrometer for seawater. Malamas *et al.* [46] made a similar study with 8-quinolinol immobilized in porous glass of on-line trace metal enrichment in water samples with added Cu, Co, Cd, Ni, Pb and Zn. The same preconcentration system was used by Devi *et al.* [47] for Cu, Cd, Mg, Hg, Zn and Pb.

Fang *et al.* [48] performed a detailed study of several on-line FIA/Flame Atomic Absorption systems for Cu, Zn, Pb and Cd preconcentration in seawater. Hartenstein *et al.* [49] used a miniature column of Chelex 100 for the determination of metals below the ppb level in tap-water and rain run-off by FIA/ICP systems. The same column and detection system is used by Hartenstein *et al.* [50] for preconcentration of 11 heavy metals and by Milosavljevic *et al.* [51] for the determination of EDTA by flame AAS. Schulze and Elsholz [52] optimized the different parameters for the Cu enrichment by means of a Chelex 100 ion exchange column inserted in the injection valve of a FIA/AAS system.

Kumamaru *et al.* [53] describe an economical on-line preconcentration system with a chelating resin column for Cd determination in biological reference materials and waste-water samples, and Bisouth *et al.* [54] describe the design and operation of a simple manifold for the preconcentration of lead and its detection by FAAS. A minicolumn of Muromac A-1 is used by Hirata *et al.* [55] for on-line preconcentration of Al, Cr, Fe, Ti and V and determination with an FIA/AES/ICP system. An interference study of Mg, Mn and Al is performed. The same resin was used for the determination of Cd and Cu in several standard reference materials with AAS detection. A study of pH dependence has also been carried out [56]. Practical considerations in the design of on-line preconcentration columns for FIA-Atomic Spectrometric systems and their application to Co determination in water was reported by Fang *et al.* [57].

Zhang *et al.* [58] determined lead in different water samples (potable, river, ground water) with on-line preconcentration by an activated alumina column, and studied possible interferences due to Ca, Mg, Na and K. The use of alternative organic-based material, including resin 122 [59], immobilized quinolin-8-ol [60,61] and tri(pyridylmethil)ethylene-diamine [62] has met with considerable success for this purpose. This column was also used for divalent and trivalent metals in water and phosphorus in steel [63]. On-line preconcentration on a Dowex A-1 resin is carried out by Wada *et al.* [64] for Ca and Mg determination in brine using spectrophotometric detection. The addition of triethanolamine and 2,3-dimercapto-1-propanol removes the possible interferences from Fe, Al, Cu, Zn and Mg.

Traces of uranium in geological samples were spectrophotometrically determined with on-line separation and preconcentration using a Levextrel C1-5209 resin [65]. Anderson and McLeod [66] used an Amberlite XA 743 boron-specific resin in a FIA/ICP system and studied the Fe interference in the determination of boron in synthetic steel samples. Ultratrace analysis of selenium and bismuth in waters (tap waters, snow waters, mineral water and soil water extracts) was performed by Zhang *et al.* [67] using a D-201 and CPG-8Q chelating ion exchanger combined with flow injection hydride generation-AAS. An exhaustive interference study is enclosed.

Yamane [68] described a method based on a catalytic detection and on-line ion-exchange separation for the determination of low levels of Mn, Cu and Fe in natural waters. Metals in the effluent were monitored spectrometrically using a colour-forming reaction with photocatechnic acid. Stewart *et al.* [69] designed an on-line ion-pair extraction for carboxylic acid analysis by spectrophotometric detection.

Yoza and co-workers [70,71] studied the separation and determination of inorganic phosphates by applying an ion-exchange column in the flow-injection manifold. Hirai *et al.* [72], Yokoyama and Tarutati [73], and Kuroda *et al.* [74] have determined silicates through the formation of 12-molybdosilicic acid. Narusawa and Hashimoto [75] proposed the separation of silicate, phosphate and arsenate with an anion exchange column installed in the flow-injection manifold. The simultaneous determination of the same analytes with on-line TSK-gel SAX column separation was described by Narusawa [76] in standard solutions. The same column was used by Narusawa *et al.* [77] for simultaneous determination of silicon and phosphorus in ashes of biological standard materials (bovine liver, chlorella and pepper bush). Devi and Townshend [78] proposed a method for nitrate determination based on the desplacement of thiocyanate from an anion-exchange minicolumn and further detection after its reaction with Fe(III). A combined suppressor column of lead and silver form resin for removing interferent ions and a Cd reductor column were used when nitrates were determined in tap water.

The determination of selenium in copper alloys and nickel sponges was reported by Ikeda [79]. In this work, an on-line removal of transition metal interferences was accomplished by a minicolumn of a chelating resin with iminodiacetate groups before an AAS detection. Kamson and Townshend [80] used an anion exchange resin column for on-line removal of interferences from phosphate and sulphate in the determination of calcium. Other works using an on-line trace enrichment using FIA system and AAS or ICP detection are described for the determination of nickel [81], non-metals [82], mercury in waters [83], Cu and Co in silicate rocks [84], heavy metals in seawater [85] and Cu and Zn in water [86].

Many reviews have been published on this subject [87–90].

On-line separations using ion chromatography coupled with an FIA system have been described in the literature for $F^-$ and $Ac^-$ in fermented products [91], and for Co at pg/ml levels [92].

Luque de Castro [16] indicated that FIA allows for the incorporation into its configuration of very different separation techniques, such as gas–liquid, gas–solid, liquid–solid, liquid–liquid, etc., to solve many common problems found in analytical chemistry. These separation techniques, coupled on-line with FIA systems, have gained significance in recent years [93]. Gallego *et al.* [94] indicated that over 50 papers have been published on this topic. The most extensively used extraction techniques are dialysis and liquid–liquid extraction.

Dialysis has been scarcely used in analytical chemistry because it has been considered to be a slow and not very well defined process. This may be true for equilibrium dialysis or batch-wise operation but not for linear dialysis in a flow system [95]. Flow-through dialysers are extremely useful components in analytical flow systems, particularly in manifolds for flow injection analysis.

Martins *et al.* [96] studied the effect of ionic strength, pH and complexing ligands on the on-line dialysis of metal ions, particularly Zn, using an FIA system. Theoretical studies with different dialysis membranes for this ion were reported by Bernhardsson *et al.* [97]. Gorton and Ogren [98] described the properties of a combined system including an FIA enzymatic technique for glucose and urea in serum and an on-line dialyser for removing proteins and other interfering substances from the sample. The enzymatic determination of galactose in milk and lactose in urine using an on-line dialysis and amperometric detection was reported by Lundback and Olsson [99]. Interfering macromolecules were removed by dialysis in the enzymatic determination of *D*-galactose in serum [100]. Ethanol in whole blood has been spectrophotometrically determined by Maeder *et al.* [101] using an FIA system coupled with an on-line immobilized alcohol dehydrogenase reactor and an on-line dialyser.

Many authors [102–106] have described theoretical studies around the on-line liquid–liquid extraction coupled with FIA systems. A determination of anionic surfactants in waste water with an automatic continuous liquid–liquid extraction was reported by Gallego *et al.* [94]. The concentration of anionic surfactants is determined indirectly by measurement of the copper present in the organic layer by AAS. A study of several extraction solvents were carried out by Motomizu *et al.* [107] for the spectrophotometric determination of anionic surfactants in river water using a poly(tetrafluoroethylene) porous membrane to separate the organic phase. Koizumi and Suzuki [108] proposed an FIA method for the determination of total aliphatic amines in foods and alcoholic beverages, which employs a combination of an on-line liquid–liquid extraction and a PTFE membrane phase separator. A method for the determination of polyphenols in olive-oils using a flow injection/liquid–liquid extraction system has been proposed by Luque de Castro *et al.* [109]. Phenol in waste water streams at low and sub ppm levels was determined by Melcher *et al.* [110] using a flow injection system with on-line extraction of the analyte and on-line liquid chromatographic detection. Curran and Marden [111] described an FIA system with on-line extraction of benzyl alcohol from water into carbon tetrachloride; in this work, 'Gore-Tex' tubing proved to be a durable membrane separator. Bitterness in beer was automatically determined by Schick and Switala [112] – the procedure was based upon an on-line extraction of acidifed beer with iso-octane using a Micro Phase Separator and the measure of optical density of the organic phase at 275 nm. Two continuous flow solvent extraction systems using IBMK were described by Atallah *et al.* [113] for the determination of trace amounts of uranium in nuclear waste reprocessing solutions with spectrophotometric detection.

Gas-diffusion membranes are also widely used on-line in FIA systems. Van der Linden [95] has used them for the determination of ammonia, carbon dioxide, cyanide and sulphur dioxide. Cristova and van der Linden [114] have shown that hydrophilic microporous polypropylene membranes can be used in the determination of water content in organic solvents. Sullivan *et al.* [115] described a manifold for the determination of sulphur dioxide in wines using malachite green and gas-diffusion membranes. This kind of separation has been primarily used in the determination of ammonia [116–118]. Farran *et al.* [119] developed an on-line extraction system by means of a completely continuous flow analysis located prior to the liquid chromatographic column for the organophosphorus pesticide determination with UV and mass spectrometry detection. Recently Fang *et al.* [120] reviewed flow injection systems involving on-line separation and preconcentration by gas-diffusion, ion-exchange, and liquid–liquid extractions, and different applications are discussed.

Considering the great analytical interest in speciation studies, the development of an inexpensive on-line procedure for redox reactions using flow injection analysis is desirable and very useful [121,122)]. An automated flow-injection system for multispeciation using a microprocessor is described by Ruz *et al.* [123]. De Andrade *et al.* [124] propose an on-line oxidation of Cr(III) to Cr(VI) before the spectrometric determination of total chromium in standard solutions. Ce(IV) and peroxydisulphate ions were tested as possible on-line oxidants, the former being more convenient under the dynamic conditions of FIA. A silver reductor minicolumn is used by Faizullah and Townshend [125] in a flow injection system for reduction of copper(II) to copper(I) using bathocuproine disulphonic acid; spectrophotometric determination was used and no interferences were found. Al-Sowdani and Townshend [126] describe a method for europium determination after on-line reduction with Zn minicolumn and either indirect spectrophotometric determination using iron(III) and 1,10-phenanthroline or spectrofluorimetric using cerium(IV). Indirect potentiometric flow-injection determination of silver was carried out by on-line destruction of the thiosulphate present in photographic fixing solutions [127]. Other authors have described the on-line reduction columns in a

FIA system for nitrate and nitrite determination, especially in waters [128], soil extracts [129] and vegetable tissues [130].

The automation of preliminary operations involving solid samples, or liquid samples containing suspended solids, is not very easy. Few reports cover the on-line dissolution procedures in FIA systems. Bergamin *et al.* [131] reported on a method for the electrolytic dissolution of steel in a few seconds and its direct pass as dissolved material in a flow injection manifold for the spectrometric Al determination. The same method was also used for molybdenum determination [132]. A rapid determination of total phosphorus in industrial waste waters, including a capillary digestor, was described by Aoyagi *et al.* [133]. The method was based on peroxodisulphate digestion using a heated capillary tube containing a platinum wire, and subsequent spectrophotometric measurement of phosphate in a solution of ammonium molybdate containing malachite green. Tablets of vitamin C were dissolved on-line in an FIA system and this vitamin was determined photometrically with cloramine-T in the presence of a starch-potassium iodide solution [134].

Microwave ovens are an alternative for solving sample dissolving problems [135]. The microwave oven was used in the rapid sample decomposition of slurries for determination of Pb, Cd and Mn by using a closed flow system and AAS detection [136].

Other on-line sample pretreatments using separation processes based on liquid-gas interfaces, such as distillation [137–139] and filtration [140,141], have been described in the literature. De Andrade *et al.* [122] used an on-line complex formation for the spectrophotometric determination of Cr(VI). The on-line generation of $Br^-$ and its use in on-line bromation of organic compounds was described by Fogg *et al.* [3]. On-line formation of iodine from iodate, iodide and hydrogen ion and its spectrophotometric detection using normal and reversed FIA was developed by Fogg *et al.* [142].

On-line derivatization has been coupled with FIA systems and applied to chloride determination [143], bioanalysis in blood plasma [144], enantiomers drugs and other nucleophiles [145], secondary amines in non-aqueous solutions [146] and amino acid determination with on-line hydrolysis of proteins [147].

Theoretical studies about on-line dilution using flow injection systems for inductively coupled plasma atomic emission-mass spectrometry detectors has been achieved by Israel *et al.* [148]. Experimental applications for on-line dilution of samples using flow injection systems have been referred to the literature [149–152], and manifolds for this task have been described and evaluated [24,153,154].

Finally, flow injection systems involving enzymatic reactions for on-line conversion of the analyte in a measurable product have been designed by different authors [106,155–159].

### FIA/on-line process monitoring

The control and optimization of a fermentation requires on-line information about the biological processes involved. There is a need for on-line measurement of such chemical process variables as the concentration of substrates, ions and the product of interest, to establish a more precise feedback for a better control [160].

The use of automatic on-line systems for monitoring fermentation processes has been widely cited in the literature [11,161–165]; however, these authors have not mentioned FIA by name, but they talk about autoanalysers, especially those based on air-segmented flow [166] and which are commercially available [167,168]. Air-segmented continuous-flow automatic analysers, flow injection analysers and on-line HPLC systems for monitoring and control of biotechnological production processes have been discussed by Schügerl [169]. The application of microbiological sensors in continuous monitoring of fermentation processes has been reviewed by Karube *et al.* [170]. Gas detectors in biotechnological processes have been mentioned by Mandenius [171], and immobilization techniques on oxirane polymers for on-line FIA in bioindustrial chemistry were described by Scheper *et al.* [172].

The on-line determination of enzymes in biotechnological processes is an important factor with regard to process development and optimization. At present, the most common enzymes are determined off-line in the laboratory after withdrawal of a separate sample. Wet chemical methods dominate in this field, mainly because enzymes have to be measured according to the reaction schemes which are catalysed by them. With FIA it should easy to develop automatically operated enzyme determination procedures, based on reaction schemes, which can be used for fast and efficient process monitoring. The current status of on-line enzyme analysis using flow injection techniques has been reviewed by Kroner [173]. The author described a recently developed sampling module based on membrane filtration, and its application to on-line enzyme determination examples in real fermentations.

The measurement of enzyme activity has been used as a direct control parameter for an optimal fermentation [174]. Most enzyme assays can be automated for on-line analysis by means of flow injection techniques [175]. Kroner and Kula [176] described a continuous flow analysis for the evaluation of alkaline protease during a fermentation process using a membrane separation and continuous sampling from the bioreactor prior to analysis (see table). The feasibility of on-line measurement during the fermentation of extracellular hydrolases (proteases or glucosidases) on a 30 and 70 l pilot scale has been demonstrated. The change in the concentration of the solutes caused by the enzyme activity was monitored spectrometrically. Equipment for on-line monitoring of formate dehydrogenase and leucine dehydrogenase in the control of enzyme-production processes was described by

*Features of FIA/on-line process monitoring*

| Species | Detection | Vi | Detection limit | RSD (%) | Linear range |
|---|---|---|---|---|---|
| Extracellular hydrolases | Spectrophot. | | | 2 | 50–500 pE ml$^{-1}$ |
| Enzymes | Spectrophot. | | | >11 IU ml$^{-1}$ | |
| Total glucose | Amp. | | | | 10 $\mu$M–0.6 mM |
| Glucose | | | | 3 | 0·20 g l$^{-1}$ |
| Microorganisms | Electrochemical | | | | |
| Intracellular ATP | L. | | <3% | | 1 mM–10 $\mu$M |
| Lactic acid | Fibre optic biosensor | | | | 0–50 mM |
| Glucose | C.L. | 50 $\mu$l | 5 mg l$^{-1}$ | <1 | 10 mg l$^{-1}$–2 g l$^{-1}$ |
| Lactic acid | C.L. | 25 $\mu$l | 50 mg l$^{-1}$ | 2 | 0·1 g l$^{-1}$–2 g l$^{-1}$ |
| Protein | Spectrophot. | 50 $\mu$l | 0·2 g l$^{-1}$ | 2 | 0·2 g l$^{-1}$–8 g l$^{-1}$ |
| Optical density | Spectrophot. | 8 $\mu$l | — | — | — |
| | | 100 $\mu$l | 0·1–40 mg l$^{-1}$ | <2% | |
| | | | | 0·1–0·25 mg ml$^{-1}$ | |
| Protein | Spectrophot. | | | | |
| | | 35 $\mu$l | 1 $\mu$g ml$^{-1}$–0·8 mg ml$^{-1}$ | 4·5–5 | |
| Extracellular Proteins | Spectrophot. | 108 $\mu$l | | 0·86 | 0·1–2·0 g l$^{-1}$ |
| L-phenylalanine | F. | 2 $\mu$l | 0·01 mM | 1·6 (0·1 mM) | 0·1–1·2 mM<br>1–30 mM |
| Urea | Fibre optic sensor | 50 $\mu$l | | 1·10 | |
| Monoclonal Antibody | F. | | | | |
| Sulphate | Turbidimetric | | 30 mg l$^{-1}$ | 2 | <200 mg l$^{-1}$ |
| Chloride | Photometric | 60 $\mu$l | | — | |
| Phosphate | Photometric | 120 $\mu$l | | 0·5–5 mg l$^{-1}$ | |
| Fluoride | Potentiometric | 60 $\mu$l | | — | |
| Inorg. mercury | C.V.A.A. | | 0·02 $\mu$g ml$^{-1}$ | 0·93 | >1 $\mu$g ml$^{-1}$ |
| Total mercury | | | | (6 $\mu$g ml$^{-1}$) | |
| Ammonia | Potentiometric | | $10^{-6}$ M (0·02 mg l$^{-1}$) | 0·2 ($10^{-4}$ M) | 2·$10^{-5}$–$10^{-2}$ M |
| Hydrochloric acid | Potentiometric | 30 ml | | 0·6 | 9–38% |
| Gold | A.A.S. | | | 0·002–5 mg l$^{-1}$ | |
| Cyanide | F.I. Technique | | | 20–1000 mg l$^{-1}$ | |
| Sulphuric acid | | | | | |
| Acetic acid | Conductometry | 70 $\mu$l | | | |
| Carbonyl number | | | | | |

*Notes:* AAS, atomic absorption spectrophotometry; Amp, amperometry; L, luminiscence; C.L., chemiluminiscence; F, fluorimetry; C.V.A.A, cold-vapour atomic absorption; P.E., protease units; I.E., immobilized enzyme; RSD, relative standard deviation; F.I.I.A., flow injection immuno-analysis; ISE, ion-selective electrode; Vi, sample injection volume.

Recktenwald *et al.* [177], and all necessary modifications for the flow injection implementation were also reported.

The determination of glucose in starch using immobilized enzymes and amperometric detection, and its use on-line in FIA systems for monitoring the whole process, was described by Emneus and Gorton [178]. The on-line determination of glucose in fermentation processes has also been accomplished by Valera *et al.* [179]. A FIA set-up for on-line determination of micro-organisms in fermentation processes of *E. coli* was described by Ding and Schimd [180], and the results were compared with those obtained by means of the optical density determination. Haketa *et al.* [181] developed an on-line FIA system for intracellular ATP measurement in a culture fluid of yeast and *E. coli* by means of a luminescent reaction for the determination of the micro-organism cell number. The on-line determination of lactic acid using an optic fibre biosensor and flow-injection analysis was described by Dremel *et al.* [182] in a Kefir fermentation process with automatic dilution of samples.

Nielsen *et al.* [183] described an FIA system for on-line monitoring of glucose, lactic acid, protein and optical density (OD) in a fermentation process. The fermentor was a design of the authors and the control of pH, temperature and agitation was performed by an external control loop. The monitoring of glucose and lactic acid was accomplished by enzymatic reactions and chemi-luminescent detection, whereas protein was determined by the biuret assay. Both protein and OD were detected by spectrometry and the system was controlled by a personal computer. No interferences from the other components in the fermentor broth were observed. The chloramine did, however, influence the analytical signal if the time between chloramine addition to sample injection was over 5 min, but a total transport time of more than 2 min was never used.

| Sampling frequency ($h^{-1}$) | Sample | Other characteristics | Ref. |
|---|---|---|---|
| | Fermentation broth | Ultrafiltration | 176 |
| | Downstream process | | 177 |
| 15 | Starch | I.E. Electrochemical oxidation | 178 |
| | Fermentation process | | 179 |
| 12 | Fermentation process | | 180 |
| 12 | Culture fluids of yeast and *E. coli* | Ion-Pair-Extraction | 181 |
| | Kefir fermentation | I.E. | 182 |
| 120 | Lactic | I.E. | 183 |
| 120 | acid | | |
| 80 | fermentation | | |
| — | | | |
| 40–60 | | Biuret assay | 184 |
| | | Dilution unnecessary | |
| | Biotechnological processes | | |
| 40 | | Bradford assay additional dilution steps | |
| 0·5 | Frichoderma fermentation | Bicinchoninic acid protein assay | 185 |
| 9–100 | *Rhodococus* cultivation | | 186 |
| | Kidney dialysate | I.E. sensor stopped-flow | 187 |
| 3 | Hibidroma cell fermentation | I.E. | 188 |
| | | F.I.I.A. | |
| 60 | Water | Reverse FIA | 41 |
| | Tap water | Modified | 39 |
| | Surface water | Reverse FIA | |
| | Tap water | ISE fluoride | |
| 20 | Waste water | Chemical reduction | 189 |
| | Water | | |
| 30 | Fresh water streams | ISE | 190 |
| 60 | Concentrated hydrochloric acid | ISE | 150 |
| | Productions plants | | |
| 60 | | AAS using electrothermal atomization | 192 |
| | Phenol | Non-aqueous media | 193 |
| | Acetone process stream | | |
| | Alcohol matrix | | |

Other methods for on-line protein determination in fermentation processes by FIA have also been described. In one of them [184], the sample was automatically injected by means of a sampling device in an FIA set-up coupled with the bioreactor at intervals of 6 min. Acceptable standard deviations were obtained using the Bradford and biuret protein assays and the reliability of both methods was discussed. Other authors [185] have adapted the bicinchoninic acid (BCA) protein assay to flow injection analysis for the on-line monitoring of the production of extracellular cellulases of trichoderma species in continuously operated fermentations. A good correlation between manual and FIA-determined values for most of the fermentation time was observed. The system, calibration and timing were controlled by computer. Interferences caused by reducing sugars, yeast extract, antifoamers and metabolites excreted by the organism cultivated can be eliminated by calculation if the concentration remains reasonably constant.

A timer-controlled flow-injection system coupled with a commercial sampling device (BIOPEM) for monitoring the concentration of the inductor *L*-phenylalanine in the fermentation of *Rhodoccocus sp.* is described by Nalbach *et al.* [186]. The *o*-phthaldialdehyde (OPA) assay and fluorimetric detection were used. Interferences from primary amines, amino acids and ammonium were present. Actually, the method was restricted only when one amino acid and small amounts of the other amino groups were present. Thommen *et al.* [25] give examples for the determination of aromatic amines with *in situ* diazotation and electronic dilution using colorimetric detection. The same authors described a system for the determination of remanent glucose in fermentation processes with photometric detection. Time-cycles are 120 s and 90 s, respectively. Yèrian *et al.* [187] developed and evaluated an immobilized urease sensor and applied it to continuous on-line analysis of urea in kidney dialysated fluid.

A flow injection immunoanalysis (FIIA) system connected via a sterile sampling unit to a continuous bioreactor was used by Stocklein *et al.* [188] for the on-line monitoring of monoclonal antibodies in the course of a hybridoma cell fermentation. Mouse IgG and rabbit-anti-mouse IgG immobilized in BioMag particles were used for an antigen inhibition assay and sandwich assay, respectively. The product of the enzymatic indicator was measured fluorometrically.

An on-line automated system using a reversed flow analysis for sulphate monitoring in effluent water streams and alternating reagent injection was reported by van Staden [41]. The accuracy of the proposed system was tested by comparing the results of 10 effluent water samples with those obtained by a normal flow-injection and a standard automated segmented method. The interferents, such as organic substances, suspended soils and colour, were removed by using a filtration technique. Experimental conditions for the photometric and potentiometric determination of chloride and fluoride in tap water and phosphate in surface water were given by Frenzel [39]. The reverse FIA method used permitted a fast calibration, a removal of matrix effects and an increase of confidence. An automated continuous monitoring system for the determination of inorganic and total mercury in waste waters by flow-injection analysis, followed by cold-vapour atomic absorption spectrometry, was described by Birnie [189]. The method used a typical manifold, where digestion and reduction of the sample take place. Mercury was removed by aeration from the flowing stream in a specially designed air-liquid separator. Alegret *et al.* [190] constructed an autonomous monitor prototype for the on-line determination of ammonia in water treatment plants and freshwater streams. A specially constructed ammonium electrode was used in conjunction with a gas-diffusion chamber. The membrane performance was studied by choosing a millipore GV HP09050 poly-(vinyldene)-difluoride membrane because it possessed high transfer rate and physical resistance and no cross-contamination.

A rapid on-line automated system for the determination of the HCl content in concentrated hydrochloric acid from production plants by flow injection potentiometry was described by van Staden [150]. An automated prevalve dilution technique and a home-made selective electrode as detector were used. The results are compared with those obtained by the classical Volhard method. Prop *et al.* [191] developed an automated system for the photometric determination of several chemical constituents, such as nitrate, phosphate, chloride and glucose; they also offered the possibility of on-line calibration and evaluation of a great number of samples by means of a new software package. A method for the on-line determination of gold and cyanide in solutions resulting from the extraction and recovery of gold in industrial plants was developed by Robert *et al.* [192]. Finally, analytical methods for three non-aqueous processes were described by Schick and Karges [193]: sulphuric acid determination in phenol, acetic acid determination in acetone process stream, and the determination of carbonyl numbers in a long-chain alcohol matrix. These methods were tested on an on-line process analyser which was capable of analysing up to six streams. The instrument can carry out a continuous, automated operation for up to 1 week, after which reagents and standards need to be replaced.

### Final comments

FIA will allow filtration, dilution and calibration to be easily incorporated in a single instrument. In addition FIA does not subject the probe to continuous contact with harsh or corrosive samples [194]. In spite of the advantageous aspects of FIA for on-line control process [195], the number of applications proposed so far is extraordinarily low given the technique's potential. Until the mid-1980s hardly any references can be found to the industrial applications of FIA for real continuous monitoring or control process. Only recently have applications been reported, and these are particularly in the fields of water quality surveillance and biotechnology.

The use of membranes for dialysis, gas diffusion and other separation and preconcentration techniques deserves more attention, particularly for on-line process analysis. It offers a very elegant way of avoiding interferences from many compounds and protects the detector from undesirable contamination. The advantages of the on-line coupling of FIA with separation techniques have been demonstrated in a great number of applications, both in processes and in on-line control.

Trends in the field of process monitoring are mainly directed to the design and commercialization of modular devices capable of analysing several parameters on-line. The automatic regulation of processes can also be accomplished; examples of this assertion are the automatic continuous-flow physicochemical analyser for liquids, commercialized by Mesuralp (France), the 436/O2 Ammonia Monitor, developed by Morgan Moore Engineering Ltd (UK), and the automatic detector and counter for coliform bacteria in water, commercialized by Environment S.A. (France).

### Acknowledgement

The financial support of the CICYT PA 0285 is gratefully acknowledged.

### References

1. Karlberg, B. and Pacey, G. E., *Flow Injection Analysis. A Practical Guide* (Elsevier Science Publishers, The Netherlands, 1989).
2. Valcarcel, M. and Luque de Castro, M. D., *Journal of Chromatography*, **393** (1987), 3.
3. Fogg, A. G., Ali, Md. A. and Abdalla, M. A., *Analyst*, **108** (1983), 840.
4. Callis, J. B., Illman, D. L. and Kowalski, B. R., *Analytical Chemistry*, **59** (1987), 624A.
5. Bailey, S. J., *Control Engineering* (January 1982).
6. Bailey, S. J., *Control Engineering* (August 1983).
7. Clarke, J. R. P., *Analytica Chimica Acta*, **190** (1986), 1.
8. Worsfold, P. J., *Process Monitoring by FIA. Analyticon 89* (26–28 September 1989, London).
9. Ruzicka, J., *Analytica Chimica Acta*, **190** (1986), 155.
10. Nichols, G., *On-Line Process Analyzers* (John Wiley & Sons, New York, 1988).
11. Clevett, K. J., *Process Analyzer Technology* (John Wiley & Sons, New York, 1986).
12. Reeves, P., *Analytica Chimica Acta*, **190** (1986), 45.

13. Olsson, L., Mandenius, C. F. and Hakanson, H., *Analytica Chimica Acta*, **224** (1989), 31.
14. Huskins, D. J., *Quality Measuring Instruments in On-line Process Analysis* (Horwood, Chichester, 1982).
15. van dei Linden, W. E., *Analytica Chimica Acta*, **179** (1986), 91.
16. Luque de Castro, M. D., *Journal of Automatic Chemistry*, **8** (1986), 56.
17. Furman, W. B., *Continuous Flow Analysis: Theory and Practice* (Marcel Dekker, New York, 1976).
18. Coakley, W. A., *Handbook of Automatic Analysis. Continuous Flow Techniques* (Marcel Dekker, New York, 1982).
19. Valcarcel, M. and Luque de Castro, M. D., *Flow Injection Analysis: Principles and Applications* (Ellis Horwood, Chichester, 1985).
20. Goto, M., *Trends in Analytical Chemistry*, **2** (1983), 92.
21. Ruzicka, J., *Fresenius Z. Analytical Chemistry*, **329** (1988), 653.
22. Gisin, M. and Thommen, C., *Trends in Analytical Chemistry*, **8** (1989), 62.
23. Ranger, C. B., in *Automated Stream Analysis for Process Control*, Ed. Manka, D. P. (Academic Press, New York, 1982), Vol. 1, Chap. 2.
24. Moller, J., *Fresenius Z. Analytical Chemistry*, **329** (1988), 685.
25. Thommen, C., Garn, M. and Gisin, M., *Fresenius Z. Analytical Chemistry*, **329** (1988), 678.
26. Gisin, M. and Thommen, C., *Analytica Chimica Acta*, **190** (1986), 165.
27. Lazaro, F., Luque de Castro, M. D. and Valcarcel, M., *Journal of Pharmaceutical and Biomedical Analysis*, **6** (1988), 585.
28. Christian, G. D. and Ruzicka, J., *Chemical Engineering*, **95** (1988), 57.
29. Winter, B., *Gewaesserschutz, Wasser, Abwasser*, **102** (1988), 209.
30. Cornish, D. C., Jepson, G. and Smurthwaite, M. J., *Sampling Systems for Process Analyzers* (Butterworths, London, 1981).
31. Valcarcel, M. and Gallego, M., in *Flow Injection Atomic Spectroscopy*, Ed. Burguera, J. L. (Marcel Dekker, New York, 1989), p. 157.
32. Fang, Z., *Practical Spectroscopy*, **7** (1989), 103.
33. van Staden, J. F., *Practical Spectroscopy*, **7** (1989), 49.
34. van der Linden, W. E. M., *Kemi*, **15** (1989), 459.
35. Fogg, A. G., *Analyst*, **111** (1986), 859.
36. Johnson, K. S. and Petty, R. L., *Analytical Chemistry*, **54** (1982), 1185.
37. Fogg, A. G. and Bsebsu, N. K., *Analyst*, **109** (1984), 19.
38. Rios, A., Luque de Castro, M. D. and Valcarcel, M., *Analytical Chemistry*, **60** (1988), 1540.
39. Frenzel, W., *Fresenius Z. Analytical Chemistry*, **329** (1988), 668.
40. Fogg, A. G., Bsebsu, N. K. and Abdalla, M. A., *Analyst*, **107** (1982), 1462.
41. van Staden, J. F., *Fresenius Z. Analytical Chemistry*, **326** (1987), 754.
42. Gisin, M. and Jardas, Z., FACCS 11th Annual Meeting held in Philadelphia (1984), Abstr. No. 354.
43. Smith, B., Sherry, A. and Cherdak, A., FACCS 11th Annual Meeting held in Philadelphia (1984), Abstr. No. 355.
44. Petty, R. L. and Johnson, K. S., FACCS 11th Annual Meeting held in Philadelphia (1984), Abstr. No. 356.
45. Olsen, S., Pessenda, L. C. R., Ruzicka, J. and Hansen, E., *Analyst*, **108** (1983), 905.
46. Malamas, F., Bengtsson, M. and Johansson, G., *Analytica Chimica Acta*, **60** (1984), 1.
47. Devi, S., Habib, K. A. J. and Townshend, A., *Química Analítica*, **8** (1989), 159.
48. Fang, Z., Ruzicka, J. and Hansen, E. H., *Analytica Chimica Acta*, **164** (1984), 23.
49. Hartenstein, S. D., Christian, G. D. and Ruzicka, J., *Canadian Journal of Spectroscopy*, **30** (1985), 144.
50. Hartenstein, S. D., Ruzicka, J. and Christian, G. D., *Analytical Chemistry*, **57** (1985), 21.
51. Milosavljevic, E. B., Solujic, L., Hendrix, J. L. and Nelson, J. H., *Analyst*, **114** (1989), 805.
52. Schulze, G. and Elsholz, O., *Fortschr. Atomspek. Spurenanal*, **2** (1986), 261.
53. Kumamaru, T., Matsuo, H., Okamoto, Y. and Ikeda, M., *Analytica Chimica Acta*, **181** (1986), 271.
54. Bisouth, S. R., Tyson, J. F. and Stockwell, P. B., *Journal of Automatic Chemistry*, **11** (1989), 36.
55. Hirata, S., Umezaki, Y. and Ikeda, M., *Analytical Chemistry*, **58** (1986), 2602.
56. Hirata, S., Honda, K. and Kumamaru, T., *Analytica Chimica Acta*, **221** (1989), 65.
57. Fang, Z., Xu, S. and Zhang, S., *Analytica Chimica Acta*, **200** (1987), 35.
58. Zhang, Y., Riby, P., Cox, A. G., McLeod, C. W., Date, A. R. and Cheung, Y. Y., *Analyst*, **113** (1988), 125.
59. Ruz, J., Rios, A., Luque de Castro, M. D. and Valcarcel, M., *Fresenius Z. Analytical Chemistry*, **322** (1985), 499.
60. Marshall, M. A. and Mottola, H. A., *Analytical Chemistry*, **57** (1985), 729.
61. Marshall, M. A. and Mottola, H. A., *Analytica Chimica Acta*, **158** (1985), 369.
62. Bengtsson, M., Malamas, F., Torstensson, A., Regnell, O. and Johansson, G., *Mikrochimica Acta*, **III** (1985), 209.
63. McLeod, C. W., Zhang, Y., Cook, I., Cox, A., Date, A. R. and Cheung, Y. Y., *Journal of Research of the National Bureau of Standards*, **93** (1988), 462.
64. Wada, H., Asakura, K., Rattaiah, G. V., and Nakagawa, G., *Analytica Chimica Acta*, **214** (1988), 439.
65. Wu, X. and Qi, W., *Analytica Chimica Acta*, **214** (1988), 279.
66. Anderson, D. R. and McLeod, C. W., *Analytical Proceedings*, **25** (1988), 67.
67. Zhang, S., Xu, S. and Fang, Z., *Química Analítica*, **8** (1989), 191.
68. Yamane, T., *Analytical Sciences*, **2** (1986), 191.
69. Stewart, J. T., Lang, J. R. and Honigberg, I. L., *Journal of Liquid Chromatography*, **11** (1988), 3353.
70. Yoza, N., Hirano, H., Baba, Y. and Ohashi, S., *Journal of Chromatography*, **325** (1985), 385.
71. Yoza, N., Sagara, Y., Morioka, H., Handa, T., Hirano, H., Baba, Y. and Ohashi, S., *Journal of Flow Injection Analysis*, **3** (1986), 37.
72. Hirai, Y., Yokoyama, T., Yoza, N., Tarutani, T. and Ohashi, S., *Bunseki Kagaku*, **30** (1981), 350.
73. Yokoyama, T. and Tarutani, T., *Journal of Flow Injection Analysis*, **2** (1985), 30.
74. Kuroda, R., Ida, I. and Kimura, H., *Talanta*, **32** (1985), 353.
75. Narusawa, Y. and Hashimoto, T., *Chemical Letters* (1987), 1367.
76. Narusawa, Y., *Analytica Chimica Acta*, **204** (1988), 53.
77. Narusawa, Y., Katsura, T. and Kato, F., *Fresenius Z. Analytical Chemistry*, **332** (1988), 162.
78. Devi, S. and Townshend, A., *Analytica Chimica Acta*, **225** (1989), 331.
79. Ikeda, M., *Analytica Chimica Acta*, **170** (1985), 217.
80. Kamson, O. F. and Townshend, A., *Analytica Chimica Acta*, **155** (1983), 253.
81. Fang, Z., Xu, S. and Zhang, S., *Fenxi Huaxue*, **12** (1984), 997.
82. Menendez Garcia, A., Sanchez Uria, J. E.,

Sanz-Medel, A. and Cotrino Bautista, J., International Symposium on Detection in Liquid Chromatography and Flow Injection Analysis (HPLC/FIA) held in Córdoba, Spain (1989), 70.
83. Zhang, S., Xu, S. and Fang, Z., *Guangpuxue Yu Guangpu Fenxi,* **8** (1988), 39.
84. Valcarcel, M., Gallego, M. and Santelli, R. E., International Symposium on Detection in Liquid Chromatography and Flow Injection Analysis (HPLC/FIA) held in Córdoba, Spain (1989), 102.
85. Fang, Z. and Welz, B., *Journal of Analytical Atomic Spectrometry,* **4** (1989), 543.
86. Wang, X. and Barner, R. M., *Journal of Analytical Atomic Spectrometry,* **4** (1989), 509.
87. Zagatto, E. A. G., Krug, F. J., Bergamin, H., Jr. and Joergensen, S. S., *Practical Spectroscopy,* **7** (1989), 225.
88. Burguera, M., Burguera, J. L. and Pacey, G. E., *Practical Spectroscopy,* **7** (1989), 293.
89. Valcarcel, M. and Gallego, M., *Trends in Analytical Chemistry,* **8** (1989), 34.
90. Wada, Y., *Nippon Kaisui Gakkaishi,* **43** (1981), 13.
91. Esteve Juan, M. E. and Maquieira, A., International Symposium on Detection in Liquid Chromatography and Flow Injection Analysis (HPLC/FIA) held in Córdoba, Spain (1989), 67.
92. Sakai, H., Fujiwara, T., Yamamoto, N. and Kumamaru, T., *Analytica Chimica Acta,* **221** (1989), 249.
93. Karlberg, B., *Fresenius Z. Analytical Chemistry,* **329** (1988), 660.
94. Gallego, M., Silva, M. and Valcarcel, M., *Analytical Chemistry,* **58** (1986), 2265.
95. van der Linden, W. E., *Analytica Chimica Acta,* **151** (1983), 359.
96. Martins, E., Bengtsson, M. and Johansson, G., *Analytica Chimica Acta,* **169** (1985), 31.
97. Bernhardsson, B., Martins, E. and Johansson, G., *Analytica Chimica Acta,* **167** (1985), 111.
98. Gorton, L. and Ogren, L., *Analytica Chimica Acta,* **106** (1981), 45.
99. Lundback, H. and Olsson, Bo., *Analytical Letters,* **18** (1985), 871.
100. Olsson, B., Lundback, H. and Johansson, G., *Analytica Chimica Acta,* **167** (1985), 123.
101. Maeder, G., Polletier, M. and Haerdi, W., International Symposium on Detection in Liquid Chromatography and Flow Injection Analysis (HPLC/FIA) held in Córdoba, Spain (1989), 80.
102. Nekimken, H. L., Smith, B. F., Jarvinen, G. D., Peterson, E. J. and Jones, M. M., *Analytical Chemistry,* **60** (1988), 1390.
103. De Ruiter, C., Wolf, J. H., Brinkman, U. A. Th. and Frei, R. W., *Analytica Chimica Acta,* **192** (1987), 267.
104. Risinger, L., Johansson, G. and Thorneman, T., *Analytica Chimica Acta,* **224** (1989), 13.
105. Cañete, F., Rios, A., Luque de Castro, M. D. and Valcarcel, M., *Analytica Chimica Acta,* **224** (1989), 169.
106. Ruzicka, J. and Hansen, E. H., *Flow Injection Analysis* (John Wiley & Sons, New York, 1988).
107. Motomizu, S., Oshima, M. and Kuroda, T., *Analyst,* **113** (1988), 747.
108. Koizumi, H. and Suzuki, Y., *Analytical Sciences,* **4** (1988), 537.
109. Luque de Castro, M. D., Valcarcel, M., Linares, P. and Mesa, J. A. G., International Symposium on Detection in Liquid Chromatography and Flow Injection Analysis (HPLC/FIA), held in Córdoba, Spain (1989), 103.
110. Melcher, R. G., Cortes, H. J., Ngo, M. M. and Pillepich, J. L., Winter Conference on Flow Injection Analysis held in Orlando, Florida, on 5–7 January (1989).
111. Curran, D. J., and Marden, S. K., Winter Conference on Flow Injection Analysis held in Orlando, Florida, on 5–7 January (1989).
112. Schick, K. G. and Switala, K., Winter Conference on Flow Injection Analysis held in Orlando, Florida, on 5–7 January (1989).
113. Atallah, R. H., Christian, G. D. and Hartenstein, S. D., *Analyst,* **113** (1988), 463.
114. Cristova, L. M. M. and van der Linden, W. E., Unpublished results.
115. Sullivan, J. J., Hollingsworth, T. A., Wekell, M. W., Newton, R. T. and Larose, J. E., *Journal of the Association Official of Analytical Chemists,* **69** (1986), 542.
116. Pasquini, C. and Cardoso de Faria, L., *Analytica Chimica Acta,* **193** (1987), 19.
117. Nakata, R., Kawamura, T., Sakashita, H. and Nitta, A., *Analytica Chimica Acta,* **208** (1988), 81.
118. Kunnecke, W. and Schmid, R. D., International Symposium on Detection in Liquid Chromatography and Flow Injection Analysis (HPLC/FIA) held in Córdoba, Spain (1989), 97.
119. Farran, A., Cortina, J. L., de Pablo, J. and Barcelo, D., International Symposium in Liquid Chromatography and Flow Injection Analysis (HPLC/FIA) held in Córdoba, Spain (1989), 35.
120. Fang, Z., Zhu, Z., Zhang, S., Guo, L. and Sun, L., *Analytica Chimica Acta,* **214** (1988), 41.
121. Storgaad Jorgensen, S. and Regitamo, M. A. B., *Analyst,* **105** (1980), 292.
122. de Andrade, J. C., Rocha, J. C., Pasquini, C. and Baccan, N., *Analyst,* **108** (1983), 621.
123. Ruz, J., Torres, A., Rios, A., Luque de Castro, M. D. and Valcarcel, M. D., *Journal of Automatic Chemistry,* **8** (1986), 70.
124. de Andrade, J. C., Rocha, J. C. and Baccan, N., *Analyst,* **109** (1984), 645.
125. Faizullah, A. T. and Townshend, A., *Analytica Chimica Acta,* **172** (1985), 291.
126. Al-Sowdani, K. H. and Townshend, A., *Analytica Chimica Acta,* **201** (1987), 339.
127. Nyasulu, F., *Analytica Chimica Acta,* **220** (1989), 287.
128. Joubert, A. E. and van Vliet, H. R., *Frezenius Z. Analytical Chemistry,* **325** (1986), 150.
129. Karlicek, R., Dolejsova, J. and Polasek, M., *Agrochemia,* **28** (1989), 119.
130. Schaller, K., Lenhardt, R., Weber, V. and Euler, C., *Fresenius Z. Analytical Chemistry,* **329** (1988), 701.
131. Bergamin, F$^{o}$., Krug, F. J., Zagatto, E. A. G., Arruda, E. C. and Coutinho, C. A., *Analytica Chimica Acta,* **190** (1986), 177.
132. Bergamin, F$^{o}$., Krug, F. J., Reis, B. F., Nobrega, J. A., Mesquita, M. and Souza, I. G., *Analytica Chimica Acta,* 214 (1988), 397.
133. Aoyagi, M., Yasumasa, Y. and Nishida, A., *Analytica Chimica Acta,* **214** (1988), 229.
134. Dpto. Quimica Analitica. Apuntes Curso Internacional Automatización de Procesos de Control de Laboratorio held in Córdoba, Spain, on 27 February–3 March (1989).
135. Bettinelli, M., Baroni, U. and Pastorelli, N., *Analytica Chimica Acta,* **225** (1989), 159.
136. Carbonell, V., Salvador, A., de la Guardia, M., Burguera, J. L. and Burguera, M. International Symposium on Detection in Liquid Chromatography and Flow Injection Analysis (HPLC/FIA) held in Córdoba, Spain (1989), 56.
137. Pihlar, B. and Kosta, L., *Analytica Chimica Acta,* **114** (1980), 275.
138. Valcarcel, M. and Gallego, M., *Practical Spectroscopy,* **7** (1989), 157.
139. Ranger, C. B., *International Laboratory* (September 1989), 64.

140. Martinez, P., Gallego, M. and Varcarcel, M., *Analytical Chemistry,* **59** (1987), 69.
141. Mir, J. M., Mateos, A. and Castillo, J. R., International Symposium on Detection in Liquid Chromatography and Flow Injection Analysis (HPLC/FIA) held in Córdoba, Spain (1989), 89.
142. Fogg, A. G., Wang, X. and Tyson, J. F., *Analyst,* **114** (1989), 1119.
143. Tyson, J. F., Fogg, A. G. and Wang, X., *Química Analítica,* **8** (1989), 179.
144. van Opstal, M. A. J., Blauw, J. S., Holthuis, J. J. M., van Bennekon, W. P. and Bult, A., *Analytica Chimica Acta,* **202** (1987), 35.
145. Krull, I. S., Gao, C. X., Chou, T. Y., Colgan, S. T., Lin, S. and Bourgue, A. T., International Symposium on Detection in Liquid Chromatography and Flow Injection Analysis (HPLC/FIA) held in Córdoba, Spain (1989), 32.
146. Whiteside, I. R. C., Worsfold, P. J. and McKerrell, E. H., *Analytica Chimica Acta,* **204** (1988), 343.
147. Ichiba, H., Niwayama, H. and Yajima, T., *Chemical Pharmaceutical Bulletin,* **37** (1989), 3155.
148. Israel, Y., Lasztity, A. and Barnes, R. M., *Analyst,* **114** (1989), 1259.
149. Bysouth, S. R. and Tyson, J. F., *Journal of Analytical Atomic Spectrometry,* **2** (1987), 217.
150. van Staden, J. F., *Fresenius Z., Analytical Chemistry,* **328** (1987), 68.
151. Bysouth, S. R. and Tyson, J. F., *Analytical Proceedings,* **23** (1986), 412.
152. Bubnis, B. P., Winter Conference on Flow Injection Analysis held in Orlando, Florida on 5—7 January (1989).
153. Tyson, J. F. and Bysouth, S. R., *Journal of Analytical Atomic Spectrometry,* **3** (1988), 211.
154. Toei, J., *Analytical Letters,* **21** (1988), 1633.
155. Ruz, J., Lazaro, F., and Luque de Castro, M. A., *Journal of Automatic Chemistry,* **10** (1988), 15.
156. Peterson, B. A., *Analytical Letters,* **22** (1989), 83.
157. Yerian, T. D., Christian, G. D. and Ruzicka, J., *Analytica Chimica Acta,* **204** (1988), 7.
158. Gizurarson, S., *Journal of Automatic Chemistry,* **11** (1989), 87.
159. Hansen, E. H., *Analytica Chimica Acta,* **216** (1989), 257.
160. Valentini, L. and Razzano, G., in *Modelling and Control of Biotechnical Processes,* Ed. Halme, A. (Proceedings of the 1st IFAC-Workshop held in Helsinki) (Pergamon, Oxford, 1983), 253.
161. Niehoff, J., Moller, J., Hiddessen, R. and Schügerl, K., *Analytica Chimica Acta,* **190** (1986), 205.
162. Bayer, Th., Herold, Th., Hiddessen, R., and Schügerl, K., *Analytica Chimica Acta,* **190** (1986), 213.
163. Ahlmann, N., Niehoff, A., Rinas, U., Schepper, Th. and Schügerl, K., *Analytica Chimica Acta,* **190** (1986), 221.
164. Gibson, T. D. and Woodward, J. R., *Analytica Chimica Acta,* **213** (1988), 61.
165. Varcarcel, M. and Luque de Castro, M. D., *Automatic Methods of Analysis* (Elsevier Science Publishers, The Netherlands, 1988).
166. Daunert, S., Bachas, L. G., Ashcom, G. S. and Meyerhoff, M. E., *Analytical Chemistry,* **62** (1990), 314.
167. Criddle, W. J., Parry, K. W. and Jones, T. P., *Analyst,* **112** (1987), 615.
168. Mizutani, S., Iijima, S., Morikawa, M., Shimizu, K., Matsuwara, M., Ogawa, Y., Matsumoto, K. and Kobayashi, T., *Journal of Fermentation Technology,* **65** (1987), 325.
169. Schügerl, K., *Analytica Chimica Acta,* **213** (1988), 1.
170. Karube, I., Tamiya, E., Sode, K., Yokoyama, K., Kitagawa, Y., Suzuki, H. and Asano, Y., *Analytica Chimica Acta,* **213** (1988), 69.
171. Mandenius, C. F., *Analytica Chimica Acta,* **215** (1988), 71.
172. Scheper, T., Dullan, T., Hundeck, H. G., Reinhardt, B. and Schuenegerl, K., *GIT Fachz. Lab.,* **33** (1989), 799, 803, 807.
173. Kroner, K. H., *Fresenius Z. Analytical Chemistry,* **329** (1988), 718.
174. Husted, H., Kroner, K. H. and Kula, M. R., *BTF-Biotech. Forum,* **2** (1985), 57.
175. Husted, H., Kroner, K. H. and Kula, M. R., *BTF-Biotech-Forum,* **2** (1985), 56.
176. Kroner, K. H. and Kula, M. R., *Analytica Chimica Acta,* **163** (1984), 3.
177. Recktenwald, A., Kroner, K. H. and Kula, M. R., *Enzyme Microbiological Technology,* **7** (1985), 607.
178. Emneus, J. and Gorton, L., *Analytical Chemistry,* **62** (1990), 263.
179. Valero, F., Poch, M., Serra, A., Bartroli, J. and Medina, M. J., Dechema Biotechnology Conference, Vol. 2 (Bioreact, Downstream Process, Process React, Modell. Bioprocesses, 1988), 103.
180. Ding, T. and Schimd, R. D., International Symposium on Detection in Liquid Chromatography and Flow Injection Analysis (HPLC/FIA) held in Córdoba, Spain (1989), 96.
181. Haketa, Y., Motohashi, R., Kajiwara, K., Matsunaga, N. and Gouda, K., *Bunseki Kagaku,* **38** (1989), T183.
182. Dremel, B. A. A., Yang, W. and Schmid, R. D., International Symposium on Detection in Liquid Chromatography and Flow Injection Analysis (HPLC/FIA) held in Córdoba, Spain (1989), 98.
183. Nielsen, J., Nikolajssen, K. and Villadsen, J., *Biotechnology and Bioengineering,* **33** (1989), 1127.
184. Recktenwald, A., Kroner, K. H. and Kula, M. R., *Enzyme Microbiological Technology,* **7** (1985), 146.
185. Stamm, W. W., Pommerening, G., Wandrey, C. and Kula, M. R., *Enzyme Microbiological Technology,* **11** (1989), 96.
186. Nalbach, U., Schiemenz, H., Stamm, W. W., Hummel, W. and Kula, M. R., *Analytica Chimica Acta,* **213** (1988), 55.
187. Yerian, T. D., Christian, G. D. and Ruzicka, J., *Analytica Chimica Acta,* **204** (1988), 7.
188. Stocklein, W., Blasey, H., Ross, A. and Schmid, R., International Symposium in Liquid Chromatography and Flow Injection Analysis (HPLC/FIA) held in Córdoba, Spain (1989), 48.
189. Birnie, S. E., *Journal of Automatic Chemistry,* **10** (1988), 140.
190. Alegret, S., Alonso, J., Bartroli, J. and Martinez-Fabregas, E., *Analyst,* **114** (1989), 1443.
191. Prop, L. T. M., Thijssen, P. C. and van Dongen, L. G. G., *Talanta,* **32** (1985), 230.
192. Robert, R. V. D., Pohlandt-Watson, Du Plessis, H. J. and Marshall, G. D., *Gov. Rep. Announce Index,* Order No. PB89-121149, 89 (1989), 19.
193. Schick, K. G. and Karges, P., Winter Conference on Flow Injection Analysis held in Orlando, Florida, on 5–7 January (1989).
194. Riebe, M. T. and Eustace, D. J., *Analytical Chemistry,* **62** (1990), 65A.
195. Luque de Castro, M. D., *Talanta,* **36** (1989), 591.

